# Electrocardiographic patterns predict the presence of collateral circulation and in-hospital mortality in acute total left main occlusion

**DOI:** 10.1186/s12872-022-02585-x

**Published:** 2022-04-02

**Authors:** Chunwei Liu, Fan Yang, Jingxia Zhang, Yuecheng Hu, Jianyong Xiao, Mingdong Gao, Le Wang, Ximing Li, Zhigang Guo, Hongliang Cong, Yin Liu

**Affiliations:** 1 Tianjin Medical Universityy, Tianjin, China; 2 Department of Diagnostic Ultrasound, Tianjin Medical University Cancer Institute and Hospital, National Clinical Research Center of Cancer, Key Laboratory of Cancer Prevention and Therapy, Tianjin, China; 3 Department of Cardiology, Tianjin Chest Hospital, Jizhao Road, Jinnan District, Tianjin, 300051 China; 4 Department of Cardiac Surgery, Tianjin Chest Hospital, Tianjin, China

**Keywords:** Left main, Acute myocardial infarction, aVR, ECG, Collateral circulation

## Abstract

**Background:**

Data on the clinical characteristics, electrocardiogram (ECG) findings and outcomes of patients with acute myocardial infarction (AMI) due to total unprotected left main (ULM) artery occlusion is limited.

**Methods:**

Between 2009 and 2021, 44 patients with AMI due to total ULM occlusion underwent primary percutaneous coronary intervention (PCI) at our institution. The ECG, collateral circulation, clinical and procedural characteristics, and in-hospital mortality were retrospectively evaluated.

**Results:**

Twenty five patients presented with shock and 18 patients had in-hospital mortality. Nineteen patients presented with ST-segment elevation myocardial infarction (STEMI), while 25 presented with non-ST-segment elevation myocardial infarction (NSTEMI). ST-segment elevation (STE) in I and STEMI were associated with the absence of collateral circulation, while STE in aVR was associated with its presence. In the NSTEMI group, patients with STE in both aVR and aVL showed more collateral filling of the left anterior descending coronary artery (LAD) territory, while patients with STE in aVR showed more collateral filling of the LAD and the left circumflex artery territory. Compared with total ULM occlusion, patients with partial ULM obstruction presented with more STE in aVR, less STE in aVR and aVL, and less STEMI. Shock, post-PCI TIMI 0–2 flow, non-STE in aVR, STEMI, and STE in I predicted in-hospital mortality. STEMI and the absence of collateral flow were significantly associated with shock.

**Conclusions:**

STE in the precordial leads predicted the absence of collateral circulation while STE in aVR and STE in both aVR and aVL predicted different collateral filling territories in ULM occlusion. STE in I, non-STE in aVR, and STEMI predicted in-hospital mortality in these patients.

## Introduction

Acute total occlusion of the unprotected left main (ULM) is an uncommon but clinically catastrophic event. In the last two decades, several contemporary registries/cohorts (BCIS [1], AMIS [2], AOI-LMCA registry [3]) have reported poor outcomes regarding the left main culprit. Most of these data consist of both total and subtotal occlusion of ULM, with a large proportion of subtotal occlusion (thrombolysis in myocardial infarction [TIMI] 1–3). Meanwhile, very little is known about the clinical characteristics and outcomes of total occlusion of ULM (TIMI 0).

The electrocardiographic (ECG) presentation is variable in patients with total occlusion of ULM in the setting of acute myocardial infarction (AMI). Several ECG patterns have been reported to be associated with ULM occlusion [4]: ST-segment elevation (STE) starting in precordial lead V2 to V4 and continuing through lead V6 and in the lateral extremity leads I and aVL and STE in lead aVR or aVR and aVL with widespread ST-segment depressions. It is unclear why only some patients with acute ULM occlusion present with STE in the precordial leads, while others present with STE in lead aVR or aVR and aVL. Moreover, aVR ST elevation was reported to be an important adverse prognostic sign in AMI [5]; however, there has been little discussion on the relationship between ECG features and clinical prognosis in patients with acute total ULM occlusion.

Accordingly, this study surveyed the clinical characteristics, collateral circulation, and ECG features of acute total ULM occlusion (TIMI 0) and sought to explore the relationship between these factors and the clinical outcomes in these patients.

## Methods

### Study population

A retrospective cohort analysis was performed on all patients with AMI due to total ULM occlusion who underwent emergent myocardial revascularization in a single center (Tianjin Chest Hospital, Tianjin, China) between November 2009 and June 2021. The inclusion criteria were as follows: patients presenting with AMI defined according to the Fourth Universal Definition of Myocardial Infarction (2018) [6] and ULM as the culprit lesion indicated by the ECG and angiographic characteristics (visible thrombus and 100% stenosis). Patients with a history of Q-wave myocardial infarction or coronary artery bypass graft surgery were excluded. The ECG data of patients with AMI due to partial ULM obstruction (TIMI ≥ 1 and > 90% stenosis) were also collected simultaneously.

### Baseline clinical and ECG data

Baseline clinical and angiographic data were retrieved from the patients’ hospital charts. All available ECGs obtained after symptom onset and before primary percutaneous coronary intervention (PCI) were included in the analysis. The ECG closest to angiography was used for analysis when more than one ECG was available. ST elevation or depression was considered if the ST deviation at the most prominent point at the J point was > 0.1 mV as previously described. [7, 8] The ECG findings were analyzed by two independent cardiologists (YL and HLC). The ECGs were divided into two patterns according to the characteristics of STE, the ST-segment elevation myocardial infarction (STEMI) and non-STEMI (NSTEMI). STEMI pattern, acute anterior myocardial infarction or extensive anterior and high lateral myocardial infarction was defined as STE in V2 to V5 and in the lateral extremity leads I and aVL; NSTEMI pattern, STE in lead aVR only or both leads aVR and aVL with widespread ST-segment depressions, but without STE in V4-V5.

### PCI procedure and clinical outcomes

Coronary angiography was reviewed by two independent operators (JXZ and YCH) and collateral circulation was classified into four types according to the territory supplied by the collateral flow: left anterior descending coronary artery (LAD), left circumflex artery (LCX), both LAD and LCX and no collateral circulation. Primary PCI was performed using standard interventional techniques, and adjunctive pharmacological treatments were administered according to STEMI guidelines. The primary endpoint of the study was in-hospital mortality, while the long-term major adverse cardiovascular events (MACE: all-cause death, nonfatal acute myocardial infarction) were assessed as the secondary endpoint.

### Statistical analysis

Data are presented as mean ± SD for continuous variables and as the proportion of valid cases for discrete variables. Differences in the baseline clinical, procedural characteristics, and ECG features were compared between the survival and mortality groups using the unpaired t-test (for continuous variables) or the Fisher exact test (for categorical variables). Fisher’s exact test was used to evaluate the relationship between the ECG features and collateral circulation. Chi-square test was used to compare differences in ECG characteristics between total ULM occlusion and partial ULM obstruction; Survival curves were generated using the Kaplan–Meier method. Receiver operating characteristic curve (ROC) analysis was used to evaluate prognoses in patients with ULM-AMI. Statistical significance was set at *p* < 0.05. The SPSS software (version 19.0; IBM Corp., New York, NY) was used for all statistical analyses.

## Results

Between November 2009 and June 2021, 48 patients with AMI due to total ULM occlusion underwent emergent coronary angiography at our institution (one patient with in-stent restenosis and one patient with acute in-stent thrombosis in ULM). Among them, 44 had primary PCI, two underwent fibrinolytic therapy followed by immediate PCI and two underwent emergent coronary artery bypass graft after coronary angiography. Collateral circulation was not assessed in two patients who underwent LM PCI before right coronary artery (RCA) angiography. So, 44 patients had LM PCI (RCA angiography prior to LM PCI) and were included in our study. Over the same period, 11, 343 patients were treated with primary PCI for AMI, and 58 patients with partial ULM obstruction (> 90% stenosis) were enrolled for ECG analysis.

### Baseline clinical data

The baseline clinical and angiographic data of the 44 consecutive patients with acute total ULM occlusion are listed in Table 1. All patients had a right-dominant coronary circulation. Intravascular microaxial left ventricular assist devices are expensive and not available for broad implementation in our district. As such, all patients were treated with a crossover stent technique from the ULM to the LAD, while the kissing balloon technique was used in only six patients. Double-stent techniques were not used in these cases. TIMI 3 flow after reperfusion treatment in both the LAD and LCX was achieved in 24 (55%) patients. Fourteen patients had TIMI 2 flow in the distal LAD, while 6 patients had TIMI 2 flow in both LAD and LCX. The following reasons accounted for the final TIMI 2 flow: slow-flow (11/20), no-reflow (5/20), “milking-like flow” [9] resulted from high left ventricular wall tension (3/20) and coronary artery dissection (1/20).

**Table 1:** Baseline data of 44 patients with acute myocardial infarction due to total unprotected left main occlusion

	Survival group (n = 26)	Mortality group (n = 18)	*P*
age	61.1 ± 10.8	65.7 ± 11.9	0.20
Male/female	23 (89%)	16 (89%)	0.97
Hypertension	15 (58%)	11 (61%)	0.82
diabetes mellitus	7 (25%)	6 (33%)	0.54
eGFR(ml/min1.73m^2^)	60 ± 22	61 ± 25	0.90
LDL level (mmol/L)	3.36 ± 0.87	3.18 ± 0.71	0.53
Cardiogenic shock	9 (35%)	16 (89%)	0.00*
Onset-to-FMC (min)	227 ± 114	210 ± 132	0.69
Collateral perfusion	14 (54%)	4 (22%)	0.04*
Final TIMI 3 flow	18 (69%)	6 (33%)	0.02*
IABP			0.08
Positioned before PCI	2	5	
Positioned during PCI	22	12	
Mechanical ventilation	8 (31%)	9 (50%)	0.20
Thrombus aspiration	7 (27%)	5 (28%)	0.95
RCA severe stenosis	7 (27%)	4 (22%)	0.72
RCA PCI simultaneously	2 (8%)	1 (6%)	0.75
LM site			0.73
Ostium	-	-	
Body	16	12	
Bifurcation	10	6	
Antiplatelet drug			
Clopidogrel	8 (31%)	7 (39%)	0.58
Ticagrelor	18 (69%)	11 (61%)	
GP IIb/IIIa inhibitors	18 (69%)	13 (72%)	0.83

*eGFR* estimated glomerular filtration rate, *LDL* low density lipoprotein, *Onset-to-FMC* symptoms onset to first medical contact, *IABP* intra-aortic balloon pump, *PCI* percutaneous coronary intervention

*p < 0.05

### Association between ECG features and collateral circulation

**Fig. 1:**
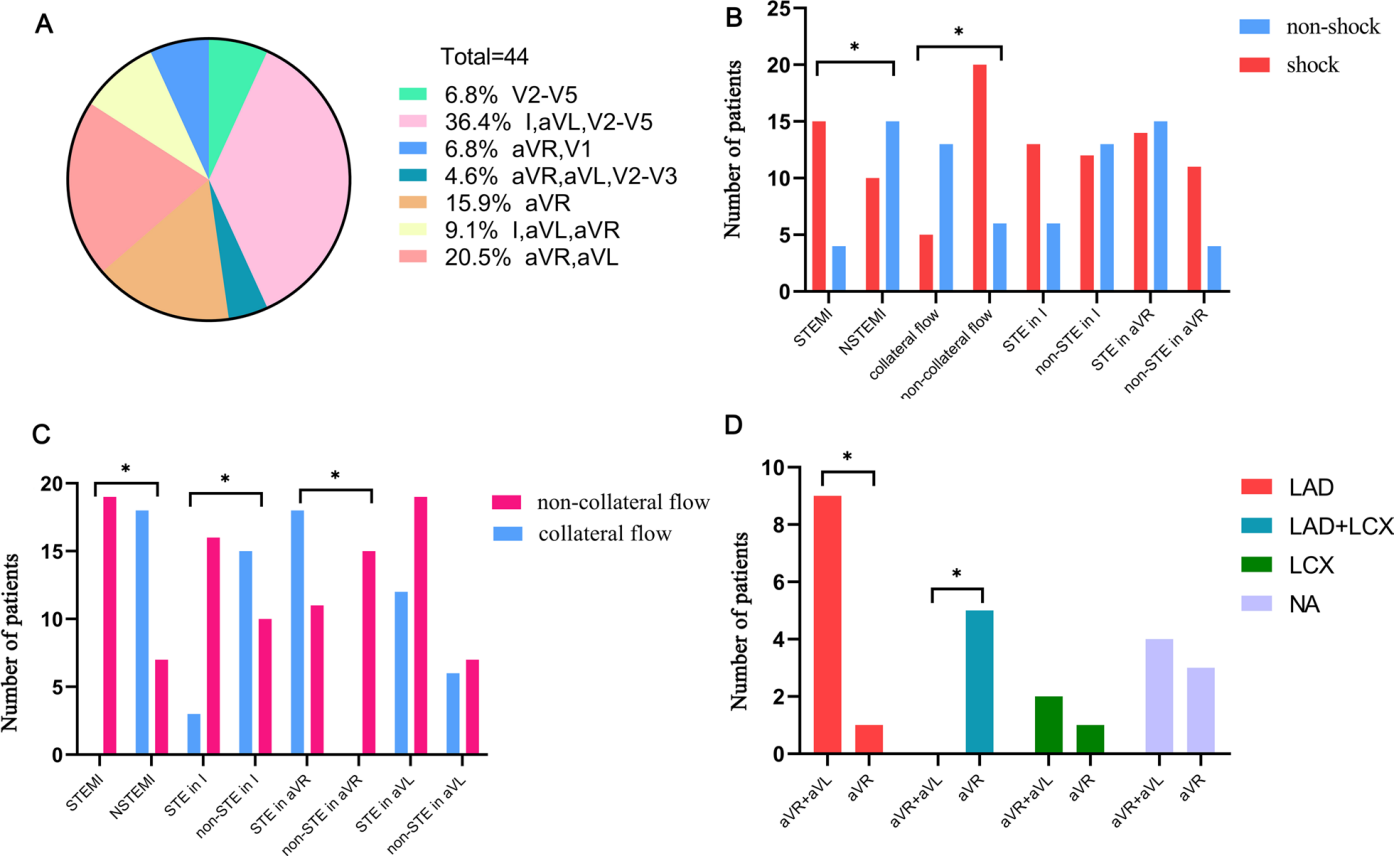
The proportion of the different STE types in ULM occlusion (**A**), the relationship between ECG characteristics and shock (**B**), The relationship between ECG characteristics and collateral circulation in ULM occlusion (**C**), the relationship between STE and collateral circulation filling territory in NSTEMI (**D**). Bars represent the population number, *p < 0.05. ECG, electrocardiogram; STE, ST-segment elevation; STEMI, ST-segment elevation myocardial infarction; NSTEMI, non-ST-segment elevation myocardial infarction

Nineteen patients presented with the STEMI pattern, while 25 patients presented with the NSTEMI pattern (Fig. 1A). Interestingly, there were three patients (only one survived) who presented with a de Winter ECG pattern, with the two evolving to ST elevation in the precordial leads within hours of presentation before any coronary intervention [10]. There was no difference in the ECG patterns among the patients stratified by age and symptom onset to first medical contact time (*p* > 0.05). STEMI and absent collateral flow were significantly associated with shock (Fig. 1B, *p* < 0.05). However, non-STE in lead aVR and STE in lead I were not associated with shock.

Eighteen patients presented with collateral circulation from the contralateral right coronary artery (RCA) (Rentrop score ≥ 1). Two patients with STE in aVL, aVR and V2-V3 had collateral flow (this ECG presentation was categorized as NSTEMI pattern due to no STE in V4-V5). Fisher’s exact test indicated that collateral flow presence/absence was associated with the characteristics of STE in ULM occlusion. STE in lead I and the STEMI pattern were associated with collateral flow absence, while STE in lead aVR was associated with its presence (Fig. 1C, *p* < 0.05). The sensitivities of STEMI, non-STE in lead aVR, and STE in lead I for collateral flow absence were 73%, 58%, and 62%, respectively. The specificities of these characteristics for collateral flow absence were 100%, 100%, and 83%, respectively.

**Fig. 2:**
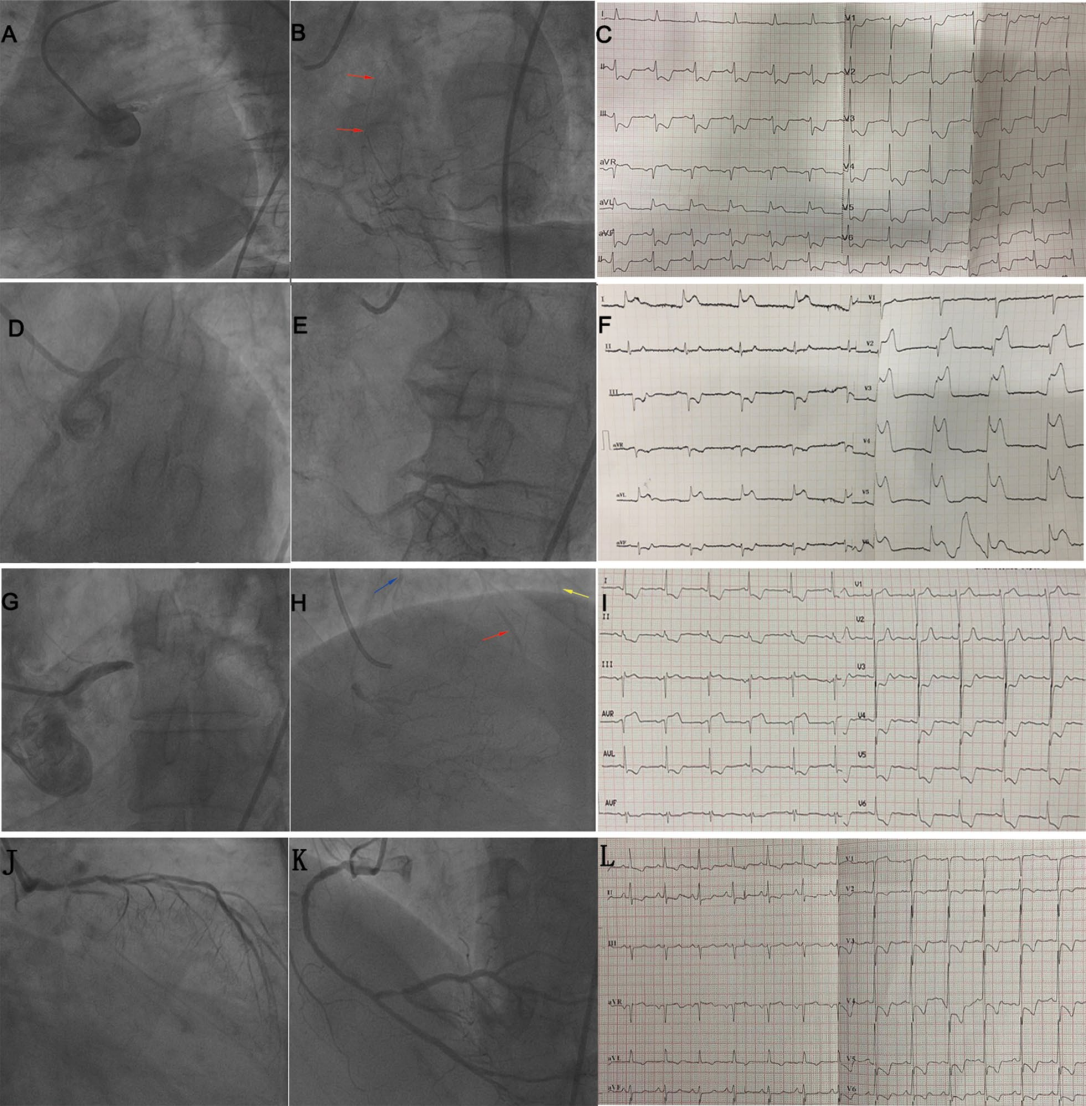
**A–C** Illustrate STE in both leads aVR and aVL in a patient with total ULM occlusion and collateral filling of LAD (red arrow). **D**–**F** illustrate STE in the precordial and lateral extremity leads in a patient with total ULM occlusion and no collateral circulation. **G**–**I** illustrate STE in lead aVR in a patient with total ULM occlusion and collateral filling of the LAD (red arrow), diagonal branch (yellow arrow), and LCX (blue arrow). **J**–**L** illustrate STE in leads avR and V1 in a patient with subtotal ULM obstruction and no collateral circulation. ULM, unprotected left main occlusion; STE, ST-segment elevation; LAD, left anterior descending coronary artery; LCX, left circumflex artery

Furthermore, Fisher exact test indicated that STE characteristics in NSTEMI were associated with collateral filling territory. Patients with STE in both leads aVR and aVL showed more collateral filling of the LAD territory, while patients with STE in lead aVR showed more collateral filling of the LAD and LCX territory (Fig. 1D, *p* < 0.05). The sensitivity of STE in lead aVR + aVL for collateral filling of the LAD territory was 90%, and the sensitivity of STE in lead aVR for collateral filling of the LAD and LCX territory was 100%. The representative ECG findings in patients with different collateral filling territories were shown in Fig. 2.

### Different ECG features between total ULM occlusion and partial ULM obstruction

**Fig. 3:**
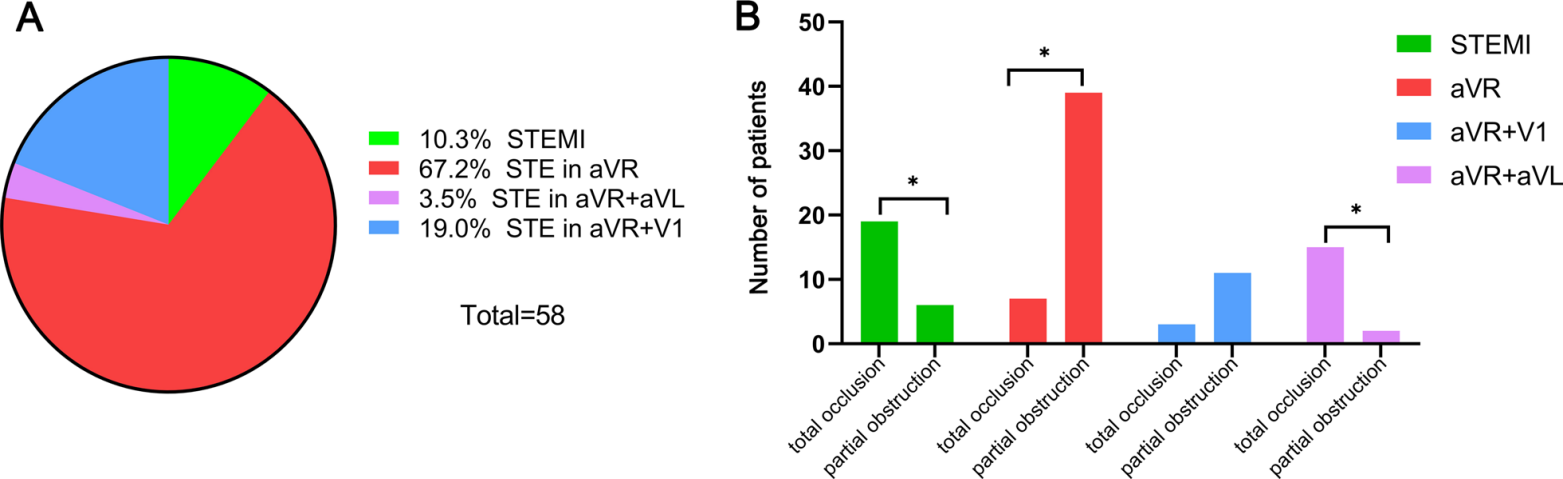
**A** Illustrates the proportion of the different STE types in partial ULM obstruction. **B** Shows the comparison of ECG characteristics between total occlusion and partial obstruction. *p < 0.05

The ECG characteristics of patients with partial ULM obstruction were shown in Fig. 3A. Compared with total occlusion, patients with partial obstruction presented with more STE in lead aVR, less STE in both leads aVR and aVL, and less STEMI (Fig. 3B, *p* < 0.05). The specificity of STE in leads aVR and aVL for total ULM occlusion was 97%, while the specificity of STE in lead aVR for partial ULM obstruction was 76%.

### In-hospital mortality and long-term outcomes

Eighteen (41%) patients died in the hospital due to cardiac rupture (3/18), refractory cardiogenic shock (10/18), or multiorgan failure (5/18). Fisher exact test indicated that cardiogenic shock, collateral flow absence, and post-PCI TIMI 0–2 flow were associated with in-hospital mortality (Table 1, *p* < 0.05). The use of an intraaortic balloon pump before PCI did not improve clinical outcomes compared with insertion during the procedure. Similarly, mechanical ventilation did not improve the clinical benefits in these patients. The ECG findings of STE in lead I, non-STE in lead aVR, and STEMI were significantly associated with in-hospital mortality (Table 2, *p* < 0.05). However, there was no difference in STE in lead aVL between the survival and mortality groups. Moreover, our study demonstrated that STE in lead I plus right bundle branch block predicted in-hospital mortality with a specificity of 96%.

**Table 2:** Comparison of electrocardiographic features of acute total unprotected left main occlusion

ECG feature	Survival group (n = 26)	Mortality group (n = 18)	*P*
STEMI	7 (27%)	12 (67%)	0.01*
NSTEMI	19 (73%)	6 (33%)	0.01*
RBBB	5 (19%)	5 (28%)	0.51
LBBB	0 (0%)	1 (6%)	0.41
LAFB	8 (31%)	6 (33%)	0.86
STE in aVL	16 (62%)	15 (83%)	0.12
STE in aVR	22 (85%)	7 (39%)	0.00*
STE in I	5 (19%)	14 (79%)	0.00*
STE in I + RBBB	1 (3.8%)	4 (22%)	0.06

*STEMI* ST-segment elevation myocardial infarction, *NSTEMI* non-ST-segment elevation myocardial infarction, *RBBB* right bundle branch block, *LBBB* left bundle branch block, *LAFB* left anterior fascicular block, *STE* ST-segment elevation

*p < 0.05

**Fig. 4:**
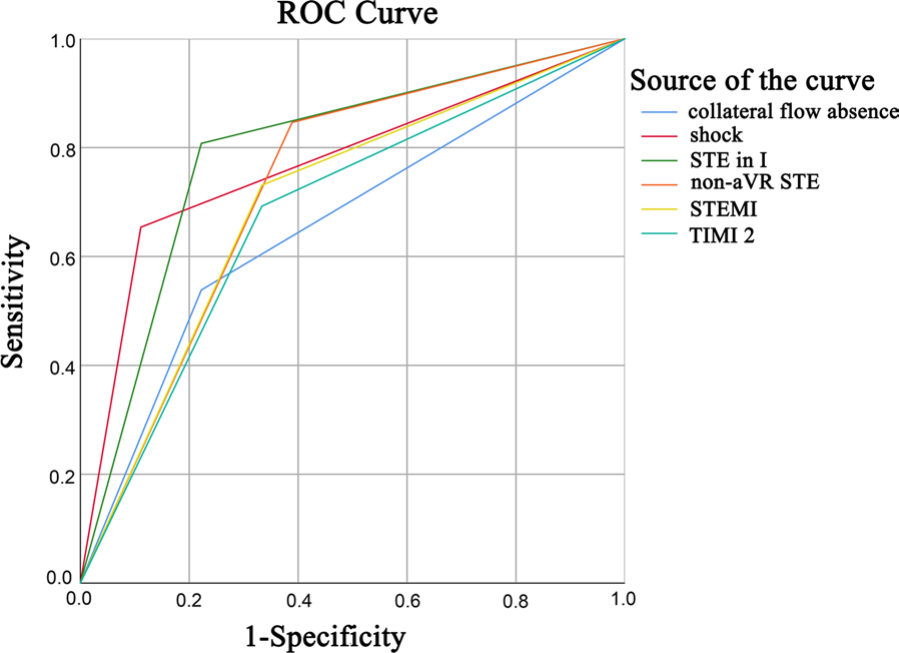
Performances of cardiogenic shock, post-percutaneous coronary intervention thrombolysis in myocardial infarction 0–2 flow, STEMI, non-STE in leads aVR, and STE in lead I in predicting in-hospital mortality, shown as receiver operating characteristic curves

We employed clinical and ECG characteristics to determine the best predictive factors in the ROC curve analyses. The areas under the ROC curve for cardiogenic shock, post-PCI TIMI 0–2 flow, collateral flow absence, STEMI, non-STE in lead aVR, and STE in lead I were 0.77, 0.68, 0.66, 0.70, 0.73, and 0.79, respectively (Fig. 4). Though STE in lead I showed a modest increase of area under ROC compared with the other factors, there was no significant differences between these risk factors.

**Fig. 5:**
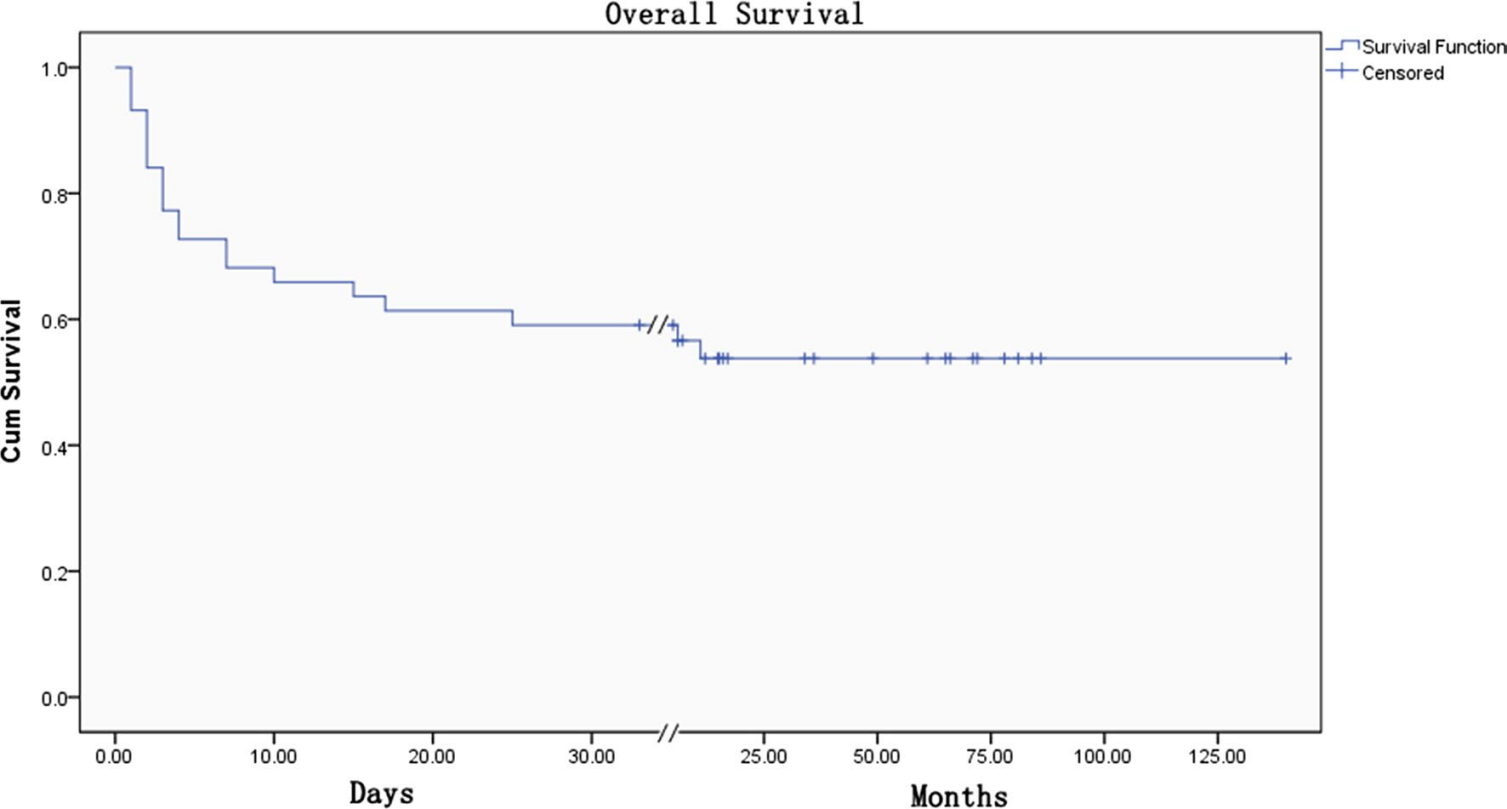
Kaplan–Meier survival curve of major adverse cardiovascular events within the median follow-up period of 45 months. The Kaplan–Meier curve estimates an overall survival of 59.1 ± 7.4% at 1 month

Within the median follow-up time of 45 months (interquartile range: 15–72 months), the overall incidence of MACE was 45%. The majority of mortality occurred within the first month post-procedure with near flattening of the survival curve afterward (Fig. 5). Only two MACEs (one reinfarction at 6 months and one death at 12 months) were reported in two patients who survived hospitalization. The probability of freedom from MACE at 1 month was 59.1 ± 7.4%.

## Discussion

To the best of our knowledge, this was the largest study on the clinical characteristics and ECG features of acute total ULM occlusion (TIMI 0). Many previous studies on ULM were vague about the degree of ULM stenosis and included patients with occlusion and partial obstruction, leading to non-specific ECG findings [4, 11, 12]. This study provided the first evidence that the ECG characteristics in patients with ULM occlusion are associated with collateral circulation.

There is uncertainty regarding the ECG features in patients with AMI due to ULM occlusion. Previous studies with small samples reported several different ECG patterns associated with acute ULM obstruction: STE in aVR with less STE in V1 [13], STE in both aVR and aVL [14] and STE in the precordial leads from V2 to V4 through V6 and in leads I and aVL [15]. In the last decades, STE in aVR with no STE in precordial leads in ULM occlusion was ascribed to the transmural ischemia of the basal part of the interventricular septum [13], or the hypothesis that anterolateral STE induced by LAD occlusion counteracted the anterolateral ST depression from the reciprocal changes of posterior infarction induced by LCX occlusion [16]. However, our study demonstrated that these different ECG patterns were associated with the different collateral filling territories in ULM occlusion: (1) STE in the anterior leads indicated the absence of collateral flow; (2) The collateral flow from the RCA into the LAD territory attenuated ST elevation in the anterior region, causing patients to present with STE in both leads aVR and aVL in this setting; 3. The collateral flow from the RCA into the LAD and LCX territory attenuated ST elevation in both the anterior and lateral extremity leads, leading to STE in lead avR only.

STE in lead aVR combined with diffuse ST depression (in leads II, III, aVF, and V4-V6) was considered to be representative of ULM occlusion in many previous studies [17, 18]. However, our study suggested that STE in lead aVR with widespread ST depression was more frequent in subtotal obstruction rather than in total occlusion. Furthermore, STE in both leads aVR and aVL was a more specific indicator for discriminating total ULM occlusion from partial ULM obstruction. This may be explained by the fact that LCX ischemia was seldom observed in subtotal ULM obstruction but was more often observed in total ULM occlusion in our study. The subanalysis from ATOLMA registry also suggested that STE in aVL predicted total ULM occlusion [19]. As STE in lead aVR with widespread ST depression indicates the injury current toward the right shoulder generated from global subendocardial ischemia, this ECG characteristic may be observed in two types of ULM culprit with a similar extent of subendocardial ischemia: subtotal obstruction and complete occlusion with well-developed coronary collateral circulation.

Previous studies have reported that some ECG characteristics are associated with in-hospital prognosis in ULM-related AMI. Yamaji et al. [13] reported that STE in lead aVR resulted in more mortalities in 16 cases of ULM obstruction, while Takayuki et al. [20] reported that STE in lead aVL rather than in aVR was associated with a poor prognosis in 89 of left main acute coronary syndromes. In contrast to these reports, our study demonstrated that STE in lead I and non-STE in lead aVR rather than STE in lead aVL predicted in-hospital mortality. The variations in the results may be explained by the difference in the inclusion criteria and selection bias due to small populations. Although STE ≥ 1 mm on lead aVR was associated with higher 30-day mortality in patients with AMI regardless of infarction location [5], we speculated that STE in lead aVR indicated the range of subendocardial ischemia rather than the severity of transmural infarction in the setting of total ULM occlusion since STE in lead aVR was observed in all the patients with collateral flow. Patients with anterior AMI due to proximal LAD occlusion before the first septal artery may have STE in leads I and aVL. The magnitude of STE in lead I is usually smaller than in lead aVL because the vector of the injury current is more parallel to lead aVL than to lead I [21]. In the case of total ULM occlusion without collateral flow, the injury current may be directed leftward and downward, more parallel to lead I. Therefore, STE in lead I may reflect a more severe myocardial ischemia.

Despite improvements in the PCI technique in recent years, the mortality of these patients remains high. Emergent revascularization is the only effective therapy to reduce mortality in patients with cardiogenic shock complicating AMI [22]. Two important questions about acute ULM occlusion in clinical practice need to be clarified: first, a primary PCI strategy is recommended over fibrinolysis when the estimated time to PCI is less than 120 min in STEMI patients. Unfortunately, most patients with ULM occlusion died before they were transported to the catheter lab. A previous study suggested that even very early angiography, with or without PCI performed < 2 h after fibrinolysis, is safe [23]. It remains unclear whether patients with ULM occlusion could benefit from fibrinolytic therapy at the time of first medical contact followed by immediate PCI. Second, the same pathological process may have two different ECG patterns in total ULM occlusion due to collateral flow absence/presence. Although STE in lead aVR with specific repolarization patterns is recognized as an equivalent of STEMI in the fourth universal definition of myocardial infarction (2018) [24], the current guidelines do not mention whether fibrinolysis is indicated in patients suspected to have ULM occlusion with STE in both leads aVR and aVL. It is unlikely that there will ever be a random comparative trial on this aspect.

In the current study, we found that cardiogenic shock, post-PCI TIMI 0–2 flow, and collateral perfusion absence were associated with early mortality, which is in line with previous studies [25, 26]. The prevalence of angiographic collaterals in patients with AMI undergoing primary PCI within 6 h of symptom onset was 23–36%.Well-developed coronary collateral arteries in patients with coronary artery disease mitigate myocardial infarcts and improve survival [27]. The long-term clinical outcome of patients surviving to hospital discharge was acceptable in our study, consistent with the findings of previous studies [1].

Our study had several limitations. First, this was a retrospective study with a small number of patients in a single center. Second, selection bias could have influenced the enrollment of patients with ULM occlusion because most of them had died before undergoing coronary angiography. Lastly, the number of participants in our study was too small to perform a multivariable-adjusted analysis of risk factors, so the predictive value of ECG patterns for in-hospital mortality may be limited. Further study is needed to evaluate these risk factors in LM occlusion, using a larger, prospective study.

## Conclusions

The present study clarified the association between ECG patterns and collateral filling territories in patients with total ULM occlusion: STE in the anterior leads indicated the absence of collateral circulation, STE in both leads aVR and aVL predicted the presence of collateral circulation from the RCA to the LAD territory, STE in aVR predicted the presence of collateral circulation from the RCA to both LAD and LCX territory. In-hospital mortality was associated with the presentation of cardiogenic shock, post-PCI TIMI 0–2 flow, collateral flow absence, STEMI, non-STE in lead aVR, and STE in lead I. STE in both leads aVR and aVL is a useful indicator for discriminating total ULM occlusion from partial ULM obstruction. Patients presenting with STE in both leads aVR and aVL should be recognized as high-risk individuals and have emergent coronary angiography.

## Data Availability

The datasets used and/or analyzed during the current study are available from the corresponding author on reasonable request.

## Abbreviations

ULM: Unprotected left main
ECG: Electrocardiographic
AMI: Acute myocardial infarction
TIMI: Thrombolysis in myocardial infarction
STE: ST-segment elevation
PCI: Percutaneous coronary intervention
ROC: Receiver operating characteristic curve
RBBB: Right bundle branch block
LBBB: Left bundle branch block
LAFB: Left anterior fascicular block
STEMI: ST-segment elevation myocardial infarction
NSTEMI: Non-ST-segment elevation myocardial infarction
LAD: Left anterior descending coronary artery
LCX: Left circumflex artery
RCA: Right coronary artery
